# RNA Dysmetabolism and Repeat-Associated Non-AUG Translation in Frontotemporal Lobar Degeneration/Amyotrophic Lateral Sclerosis due to *C9orf72* Hexanucleotide Repeat Expansion

**DOI:** 10.31662/jmaj.2022-0160

**Published:** 2022-12-23

**Authors:** Kohji Mori, Shiho Gotoh, Ryota Uozumi, Tesshin Miyamoto, Shoshin Akamine, Yuya Kawabe, Shinji Tagami, Manabu Ikeda

**Affiliations:** 1 Psychiatry, Osaka University Graduate School of Medicine, Suita, Japan; 2 Seifukai Ibaraki Hospital, Ibaraki, Japan; 3 Minoh Neuropsychiatric Hospital, Minoh, Japan; 4 Health and Counseling Center, Osaka University, Toyonaka, Japan

**Keywords:** Frontotemporal dementia, frontotemporal lobar degeneration, amyotrophic lateral sclerosis, C9orf72, Repeat-associated non-AUG translation, RNA metabolism

## Abstract

Neuropathological features of frontotemporal dementia and amyotrophic lateral sclerosis (ALS) due to *C9orf72* GGGGCC hexanucleotide repeat expansion include early dipeptide repeats, repeat RNA foci, and subsequent TDP-43 pathologies. Since the discovery of the repeat expansion, extensive studies have elucidated the disease mechanism of how the repeat causes neurodegeneration. In this review, we summarize our current understanding of abnormal repeat RNA metabolism and repeat-associated non-AUG translation in *C9orf72* frontotemporal lobar degeneration/ALS. For repeat RNA metabolism, we specifically focus on the role of hnRNPA3, the repeat RNA-binding protein, and the EXOSC10/RNA exosome complex, an intracellular RNA-degrading enzyme. In addition, the mechanism of repeat-associated non-AUG translation inhibition via TMPyP4, a repeat RNA-binding compound, is discussed.

## Introduction

Frontotemporal dementia (FTD) is a clinical concept of neurocognitive disorder that encompasses several groups of neurodegenerative conditions characterized by slowly progressive and characteristic alterations or deficit in behavior, executive function, and/or language ^(1)^. Recently, the term “frontotemporal lobar degeneration” (FTLD) is often used to define these conditions from a neuropathological/mechanistic aspect. FTLD is heterogeneous in accumulating proteins and is subclassified into several subgroups according to the major contents of the accumulating protein. Among these, FTLD cases with prominent tau pathology are called FTLD-tau, and cases with abundant TDP-43 accumulation are called FTLD-TDP.

These two categories account for roughly 90% of neuropathologically confirmed FTLD cases.

Although the majority of FTLD-TDP cases are sporadic, there are cases caused by genetic mutations. The most frequent genetic cause of FTLD-TDP is the hexanucleotide repeat expansion mutation in the intron of the *C9orf72* (chromosome 9 open reading frame 72) gene. In contrast to 2-23 (or up to 30) GGGGCC repeats in non-carriers, hundreds to more than a thousand tandem expanded repeats can be found in the expansion carriers. This mutation has been reported at a low frequency in Japan, although there are some reported cases ^(2), (3), (4)^. How these extended repeats cause TDP-43 protein abnormalities, neurodegeneration, and ultimately neurocognitive and motor neuron diseases has been actively debated since the identification of repeat expansion.

TDP-43 is a ubiquitously expressed multifunctional RNA-binding protein and confers splicing, RNA transports, and metabolism. Physiologically, TDP-43 is predominantly present within the nucleus and shuttles between the nucleus and cytoplasm; however, in FTLD-TDP cases including the *C9orf72* mutation, neuronal TDP-43 typically forms aberrant intracytoplasmic inclusions with a concomitant absence of TDP-43 from the nucleus. The higher TDP-43-positive inclusion load correlates well with the severity of neurodegeneration. Thus, TDP-43 appears to be more akin to a downstream executor of neuronal cell death.

## Mechanism of Repeat RNA Production in *C9orf72* FTLD/ALS

Aberrant DNA repeat expansion is a genetic cause and thus is unquestionably upstream of the pathological cascade. First, the DNA repeat is transcribed into an RNA repeat. As the expanded repeat is located in the intron (or promoter) of the *C9orf72* gene, the mature *C9orf72* RNA transcript does not contain the repeat region and thus none of mature C9ORF72 protein contains the translated repeat sequence. Then, how does the repeat cause neurodegeneration? One hypothetical mechanism is the repeat RNA toxicity. The repeat-containing region in *C9orf72* is bidirectionally (sense direction and antisense direction) transcribed; accordingly, the GGGGCC repeat RNA transcript and CCCCGG repeat RNA transcript are generated from the same DNA GGGGCC repeat expansion. These repeat RNA sequences can sequestrate RNA-binding proteins that preferentially bind to the repeated RNA sequence. These RNA/protein complexes may aggregate, and form intracellular structures called RNA foci. RNA foci are a neuropathological hallmark in *C9orf72* repeat expansion carriers and can be found not only in neurons but also in non-neuronal cells (astrocytes, microglial cells, fibroblasts, transformed lymphoblasts, etc.). Although still controversial ^(5)^, several studies have revealed a correlation between RNA foci with neurodegeneration in *C9orf72* FTLD/ALS cases ^(6), (7)^. Moreover, a zebrafish model demonstrated clear repeat RNA toxicity ^(8)^.

## Mechanism of DPR Production in *C9orf72* FTLD/ALS

The other disease hallmark of *C9orf72* FTLD/ALS cases is the accumulation of dipeptide repeat proteins (DPRs) ^(9)^. Transcribed intronic repeat RNA not only forms RNA foci but is also translated through repeat-associated non-AUG (RAN) translation ^(10)^. RAN translation can occur in all possible reading frames and thus results in the production of five different DPRs from the bidirectionally transcribed repeat RNA ^(9), (11), (12), (13), (14)^. Conventional translation requires a “start translation here” signal during ribosomal scanning from the 5'- to 3'-side of an RNA molecule. The start signal is usually an AUG (encoding methionine) initiation codon with a surrounding sequence that matches the Kozak rule well. Repeat-associated non-AUG translation (RAN translation) ^(10)^ is recognized as an unconventional translation event in which translation of the repetitive sequence occurs in the absence of an AUG initiation codon.

It is believed that there are at least two types of RAN translations: those initiated from near cognate codons (for example, one base mismatch) and those initiated from specific RNA structures such as RNA hairpins.

The start site of RAN translation in the poly-Gly-Ala (GA) reading frame of the *C9orf72* repeat expansion has been reported as a CUG codon in the 5'-flanking region of the GGGGCC repeat ^(15)^. It has also been suggested that this CUG codon-dependent RAN translation of poly-GA is involved in the production of poly-Gly-Pro (GP) and poly-Gly-Arg (GR) through translational frameshift ^(16), (17)^. Conversely, another group has ruled out the production of poly-GP and poly-GR by frameshifting ^(18)^. Additionally, the presence of an AUG-initiated upstream open reading frame (uORF) of 180 bases spanning exon 1 and intron 1 of the *C9orf72* gene located in the poly-GP reading frame was reported ^(19)^. This uORF ends just before the GGGGCC repeat and translation of the uORF suppresses RAN translation in poly-GA and poly-GP frames, adding further complexity to the regulation of *C9orf72* RAN translation.

The molecules that mediate RAN translation are still little known. RPS25, a factor previously known to be associated with Internal Ribosome Entry Site-dependent translation, was reported to activate RAN translation ^(20)^. The RNA helicase DHX36 unwinds the G-quadruplex structure of GGGGCC repeats and promotes RAN translation, whereas DDX3X conversely represses the RAN translation of GGGGCC repeats in an RNA helicase activity-dependent manner ^(21), (22)^. Cellular stress has also been reported to enhance RAN translation via the phosphorylation of eIF2α ^(15), (23)^. Double-stranded RNA-dependent protein kinase (PKR), which is activated by repeat RNA, also enhances RAN translation via the phosphorylation of eIF2α. Conversely, the antidiabetic drug metformin, a PKR inhibitor, inhibits RAN translation ^(24)^. These RAN translation regulators are attracting attention as potential target molecules for disease-modifying drugs in *C9orf72* FTLD/ALS via the inhibition of RAN translation.

## Mechanisms of DPR-mediated Neurodegeneration

The neurotoxicity of DPR generated by RAN translation has been shown in multiple disease models. The most abundant DPR poly-GA aggregates adsorb large amounts of stacked proteasomes, which impairs intracellular proteostasis ^(25)^. In contrast, poly-GP and poly-Pro-Ala (PA) have been shown to have no apparent toxicity ^(26)^.

Cellular organelles, such as the endoplasmic reticulum, lysosomes, mitochondria, and nucleus, are intracellularly compartmentalized by lipid bilayers, as described in textbooks. Unlike these classical organelles, intracellular structures, such as the nucleolus, centrosome, spliceosome, and stress granules, do not have such membrane partitions of lipid bilayers. Liquid-liquid phase separation is a mechanism commonly used in cells as a principle for ordering the formation and maintenance of such non-membranous structures. Poly-GR and poly-Pro-Arg (PR), that is, DPRs containing arginine residues within the repeat motif, preferentially disrupt the behavior of this liquid-liquid phase separation, leading to the impaired architecture and function of the membrane-less organelles, including stress granules ^(27), (28)^, chromatin ^(29)^, nuclear membrane pores, and nucleoli ^(27), (30), (31)^.

A major physiological function of nucleoli is the production of cellular translational machinery, the ribosome. Poly-GR and poly-PR accumulate on nucleoli and inhibit ribosomal (r)RNA production ^(27)^ in part through the inhibition of small nucleolar RNA maturation ^(32)^. In addition, poly-GR and poly-PR directly inhibit translation through their interaction with ribosomes ^(33), (34), (35)^. Thus, each DPR is thought to impair cellular function through different mechanisms, eventually leading to neuronal death.

## Identification of hnRNPA3, the Repeat RNA Suppressor

There is debate about whether the repeat RNA itself is neurotoxic because of the difficulty in fully separating repeat RNA toxicity from DPR toxicity ^(36)^. Even so, an association between RNA foci and the abnormal localization of TDP-43 has been noted in autopsy cases ^(6), (7)^. We previously established an *in vitro* RNA-binding assay to purify RNA-binding proteins that selectively bind to GGGGCC repeat RNA, followed by their identification by mass spectrometry ^(37)^. We then performed secondary immunohistochemical screening for these candidate proteins using the hippocampal tissue of patients with *C9orf72* FTLD/ALS and compared them with control tissue. From this, we noticed that hnRNPA3 is present in the cell nucleus in healthy tissues, whereas in tissues of patients with *C9orf72* FTLD/ALS, nuclear staining was variably lost, and hnRNPA3-positive inclusion pathology was observed in the hippocampal dentate gyrus ^(37)^.

So, how does hnRNPA3 affect the pathogenesis of *C9orf72* FTLD/ALS? To answer this question, we performed functional analysis of hnRNPA3 in the context of *C9orf72* FTLD/ALS. siRNA-mediated knockdown of hnRNPA3 in cells exogenously expressing GGGGCC repeats increased the accumulation level of GGGGCC repeat RNA. An RNA-binding domain mutant of hnRNPA3 lacked the ability to rescue the phenotype. Conversely, overexpression of hnRNPA3 decreased the expression level of repeat RNA. Thus, hnRNPA3 repressively regulates the repeat RNA expression level and this inhibits the DPR expression level. These findings were confirmed from analyses using patient-derived fibroblasts and primary cultured rat neurons, as well as from analyses of patient brains. These results indicate that hnRNPA3 suppresses repeat RNA expression levels by promoting repeat RNA metabolism. When hnRNPA3 is lost and this repression is compromised, there is a marked accumulation of repeat RNA and an increased expression level of DPR ^(38), (39)^.

## Mechanism of Repeat RNA Degradation by the RNA Exosome Complex and Its Disruption

As the expanded repeat hinders efficient transcription, the expression levels of mature *C9orf72* mRNA transcripts in cells derived from *C9orf72* mutation carriers are only about half that of those without the repeat expansion. However, repeat RNA, derived from the same RNA transcript, accumulates as RNA foci. As an explanation for this seemingly contradictory phenomenon, we considered the possibility that the degradation of abnormally elongated repeat RNAs is impaired.

As there was no prior knowledge of how GGGGCC repeat RNA is degraded in the cell, we first knocked down molecules constituting several representative RNA-degrading enzyme systems and monitored the expression level of DPR as a readout in preliminary experiments. With this, we found that EXOSC10 plays an important role in the degradation of repeat RNA ^(32)^. The RNA exosome complex is a multimeric protein complex that governs the metabolism of RNA and this EXOSC10 is a nucleolar enriched subunit of the RNA exosome complex. Interestingly, the genetic mutations of other components of the RNA exosome complex have been linked with neurodegenerative phenotypes ^(40), (41), (42)^.

The knockdown of EXOSC10 in fibroblasts derived from patients with* C9orf72* FTLD/ALS resulted in intracellular repeat RNA accumulation and increased nuclear RNA foci, suggesting that the EXOSC10/RNA exosome complex is involved in the metabolism of endogenous repeat RNA in patient-derived cells. In cells that expressed poly-GR or poly-PR through RAN translation, EXOSC10 was redistributed diffusely into the nucleus instead of being confined to the nucleolus.

Moreover, these arginine-rich DPRs inhibit endogenous EXOSC10 activity, which leads to the additional accumulation of GGGGCC repeat RNA. These results suggest that poly-GR and poly-PR inhibit the metabolism of repeat RNA by inhibiting the EXOSC10/RNA exosome complex. The accumulation of repeat RNA accelerates poly-GR and poly-PR production through RAN translation, thus further aggravating the pathological processes ^(32)^.

## Mechanism of Inhibition of RAN Translation by the Repeat RNA-binding Compound TMPyP4

The selective inhibition of RAN translation and DPR production may lead to the development of a novel therapeutic strategy ^(43), (44), (45)^. GGGGCC repeat RNA is known to adopt a strong tertiary structure called the G-quadruplex in the presence of potassium ions. TMPyP4 (5,10,15,20-Tetrakis-(N-methyl-4-pyridyl)porphine) is a type of porphyrin that has been reported to bind to the G-quadruplex of GGGGCC repeat RNA ^(46)^. We therefore investigated the effect of TMPyP4 on RAN translation of *C9orf72* GGGGCC repeats. In a cellular model, TMPyP4 inhibited DPR production by RAN translation, while sparing the repeat RNA expression level, nucleocytoplasmic distribution of repeat RNA, and global translational activity. Though an artificial insertion of the AUG initiation codon just before the repeat strongly enhanced repeat translation through conventional initiation, TMPyP4 strongly inhibited repeat translation even in the presence of the AUG initiation codon, suggesting that TMPyP4 does not specifically inhibit the non-AUG-dependent initiation of RAN translation. This finding led us to the hypothesis that TMPyP4 may inhibit the elongation step of RAN translation rather than non-AUG initiation. If elongation is inhibited, a large number of ribosomes stop on a single repeat RNA, and the complex of repeat RNA and ribosomes can be recovered in the higher-density fraction of sucrose density-gradient centrifugation of cytoplasmic cell lysate. Indeed, in cells treated with TMPyP4, more repeat RNA was found in the poly-ribosomal (highest density) fractions than in untreated cells. Furthermore, TMPyP4 and repeat RNA showed a strong interaction that was resistant to denaturing urea. The tight interaction between TMPyP4 and repeat RNA would physically inhibit ribosomal translocation. These results suggest that TMPyP4 binds tightly to GGGGCC repeat RNA and inhibits RAN translation by blocking the RAN translation elongation ^(47)^.

## Association of DPR with TDP-43 Proteinopathy

It has been pointed out that DPR and repeat RNA, as well as TDP-43 aggregates themselves, disrupt nucleocytoplasmic transport mechanisms and nuclear membrane pore function in multiple disease models ^(48), (49), (50), (51), (52)^. In particular, in a mouse model expressing 200 repeated poly-GR, poly-GR was found to cause TDP-43 aggregation in the cytoplasm via the mislocalization of nucleocytoplasmic transport factors and nuclear pore component proteins, pointing to a link between DPR and TDP-43 pathology ^(53)^.

The relationship between DPR pathology and clinical phenotypes in human patients remains unclear and requires further investigation. Although TDP-43 inclusion pathology correlates well with neurodegeneration, it has been noted that DPR inclusion pathology does not usually co-localize with TDP-43 inclusion. However, recent detailed reports have pointed to an association between poly-GR load and neurodegeneration ^(54), (55), (56)^. In addition, a small number of cases have been reported with clinical FTD and abundant DPR pathology at autopsy, but little or no TDP-43 pathology ^(57), (58), (59)^.

## Loss of Function of C9ORF72 Protein and *C9orf72* FTLD/ALS Pathology

The function of the C9ORF72 protein encoded by the *C9orf72* open reading frame was initially unknown, but its physiological function and role in *C9orf72* FTLD/ALS pathogenesis have gradually become clear. The C9ORF72 protein forms a heterotrimer complex with SMCR8 and WDR41 and functions in the autophagy/lysosome system. As abnormally expanded GGGGCC repeats reduce the efficiency of transcription by RNA polymerase, the expression of C9ORF72 protein is decreased ^(60), (61)^. Importantly, no neurodegeneration is observed in *C9orf72* knockout mice ^(62)^, but systemic inflammation, including in the brain, lymph node enlargement, splenomegaly, and shortened lifespan due to autoimmune reactions have been observed ^(63)^. Such lysosomal system dysfunction and systemic inflammation are considered to exacerbate repeat-mediated gain of toxicity ^(61), (64), (65)^.

## Therapeutic Approach and Utility of DPR as Biomarker

Several attempts are being made to develop a treatment for patients with *C9orf72* FTLD/ALS. One such approach is an antisense oligonucleotide (ASO), which effectively reduces repeat-containing transcripts ^(66)^. Another approach is to identify the RAN translation inhibitor that can selectively inhibit RAN translation, while sparing conventional translation as mentioned in the above section. The other approach includes repeat transcription inhibitors ^(67)^.

Biomarkers reflecting disease status are important for diagnosis, disease progression monitoring, and assessment of response to potential treatment. Although RAN translation is considered a rather inefficient event, DPRs can be detected with state-of-the-art highly sensitive assays in the cerebrospinal fluid (CSF) of patients or carriers with *C9orf72* mutations. Especially, poly-GP, which is relatively abundant and has less aggregation potency, could be detected in cases with *C9orf72* repeat expansion specific manner and is currently used as a target engagement biomarker to demonstrate proof of concept in drug discovery. Poly-GP levels are rather consistent in each patient in repeated measurements ^(68), (69), (70), (71), (72)^. The frequently used assay platforms are based on Meso Scale Discovery ELISA or single-molecule array ^(68)^.

Recent highly sensitive assays also enabled poly-GA and poly-GR measurements from the CSF of patients with *C9orf72* ^(69)^. CSF poly-GA and poly-GR levels did not correlate with clinical phenotypes, but a patient with *C9orf72*-ALS treated with an ASO targeting the repeat-containing C9orf72 transcript showed decreased CSF poly-GA, poly-GP, and poly-GR levels.

## Summary and Prospects

Extensive efforts to elucidate the pathogenesis of *C9orf72* FTLD/ALS have so far suggested that autophagic dysfunction due to haploinsufficiency of the C9ORF72 protein enhances primary gain of toxicities from repeat RNA and DPR. Interestingly, this mutation is known to cause psychiatric symptoms more frequently than FTD because of other genetic causes or sporadic FTD ^(73), (74), (75)^. By analyzing the disease mechanism of this particular mutation in detail, we hope to contribute to develop more accurate and early diagnosis and disease-modifying therapy. More specifically, we hope to extend the knowledge gained from the analysis of hereditary FTD to the pathophysiology of more frequent sporadic FTD.

## Article Information

This article is based on the study, which received the Medical Research Encouragement Prize of The Japan Medical Association in 2021.

### Conflicts of Interest

None

### Sources of Funding

K.M. is supported by the JSPS KAKENHI grant numbers JP20H03602, JP20H05927, and JP22K19492; JST FOREST Program under grant number JPMJFR200Z; and SENSHIN Medical Research Foundation and Takeda Science Foundation. S.G. is supported by a JSPS Research Fellowship for Young Scientists JP22J12248. S.A. is supported by KAKENHI JP21K15682. S.T. is supported by KAKENHI JP22K07559. M.I. is supported by AMED grant number JP21ek0109510h0001.

### Acknowledgement

We apologize that we could not cite all relevant original papers due to space limitations.
